# Advantages of whole-exome sequencing over immunomapping in 67 Brazilian patients with epidermolysis bullosa^☆^

**DOI:** 10.1016/j.abd.2023.07.002

**Published:** 2024-02-16

**Authors:** Samantha Vernaschi Kelmann, Bruno de Oliveira Stephan, Silvia Maria de Macedo Barbosa, Rita Tiziana Verardo Polastrini, Zilda Najjar Prado de Oliveira, Maria Cecília Rivitti-Machado, Gustavo Marquezani Spolador, Rachel Sayuri Honjo, Ken Saida, Naomichi Matsumoto, Chong Ae Kim

**Affiliations:** a Genetics Unit, Instituto da Criança, Hospital das Clínicas, Faculty of Medicine, Universidade de São Paulo, São Paulo, SP, Brazil; b Pain and Palliative Care Unit, Instituto da Criança, Hospital das Clínicas, Faculty of Medicine, Universidade de São Paulo, São Paulo, SP, Brazil; c Department of Dermatology, Hospital das Clinicas, Faculty of Medicine, Universidade de Sao Paulo, Sao Paulo, SP, Brazil; d Department of Human Genetics, Yokohama City University, Graduate School of Medicine Yokohama, Yokohama, Japan

**Keywords:** Epidermolysis bullosa, Exome, Genetics

## Abstract

**Background:**

Epidermolysis bullosa (EB) is characterized by skin fragility and blistering. In Brazil, the diagnosis is usually obtained through immunomapping, which involves a skin biopsy. Most recently, whole exome sequencing (WES) has become an important tool for the diagnosis of the subtypes of EB, providing information on prognosis as well as allowing appropriate genetic counseling for the families.

**Objective:**

To compare the results of immunomapping and molecular analysis and to describe the characteristics of a Brazilian cohort of patients with EB.

**Methods:**

Patients were submitted to clinical evaluation and WES using peripheral blood samples. WES results were compared to those obtained from immunomapping testing from skin biopsies.

**Results:**

67 patients from 60 families were classified: 47 patients with recessive dystrophic EB (DEB), 4 with dominant DEB, 15 with EB simplex (EBS), and 1 with junctional EB (JEB). Novel causative variants were: 10/60 (16%) in *COL7A1* associated with recessive DEB and 3 other variants in dominant DEB; one homozygous variant in *KRT5* and another homozygous variant in *PLEC*, both associated with EBS. Immunomapping was available for 59 of the 67 patients and the results were concordant with exome results in 37 (62%), discordant in 13 (22%), and inconclusive in 9 patients (15%).

**Study limitations:**

Even though EB is a rare disease, for statistical purposes, the number of patients evaluated by this cohort can still be considered limited; other than that, there was a significant difference between the proportion of types of EB (only one case with JEB, against more than 50 with DEB), which unfortunately represents a selection bias. Also, for a small subset of families, segregation (usually through Sanger sequencing) was not an option, usually due to deceased or unknown parent status (mostly the father).

**Conclusion:**

Although immunomapping has been useful in services where molecular studies are not available, this invasive method may provide a misdiagnosis or an inconclusive result in about 1/3 of the patients. This study shows that WES is an effective method for the diagnosis and genetic counseling of EB patients.

## Introduction

Epidermolysis Bullosa (EB) contemplates a group of genetic diseases that are characterized by severe skin fragility and recurrent blistering.^1^, ^2^ Most cases begin soon after birth or in early childhood, although the age of onset of the skin lesions is variable. Skin lesions usually occur due to minimal trauma. Since in EB, the basement membrane is fragile and easily damaged, there is constant flux and subsequent accumulation of extracellular fluid in the cleavage generated through the epidermis.

Overall, EB comprises a group of rare congenital disorders caused by pathogenic variants in nearly 20 genes related to the expression of proteins related to skin adherence, especially those located in the basement membrane zone. The prevalence is estimated to be approximately 8.22:1,000,000 and the incidence in the USA is considered to be 19.6:1,000,000 newborns, with similar numbers in other countries and apparently no major distinction between ethnicities. Over 30 different types of EB have been described, with a recent attempt to divide them into four large groups (2020): EB Simplex (EBS), Dystrophic EB (DEB), Junctional EB (JEB) and hybrid form, also known as Kindler Syndrome (KEB). Such classification is based mainly on inheritance pattern (autosomal dominant or recessive), severity of phenotype, and histological/immunomapping analysis.

In earlier times, electronic microscopic analysis, and afterward immunomapping through a skin biopsy, were the former gold standard for EB diagnosis. Even though either can identify the cutaneous level in which the cleavage of the blisters resides (intradermal or subepidermal) most of the time, both are usually unable to indicate with certainty the affected gene and, thus, the inheritance pattern of each patient. Besides that, skin biopsy is an invasive procedure (with the need for local anesthesia and subsequent suture) and when its results are inconclusive, there is often a delay and need for a second sample, which can be particularly problematic in EB patients.^3^

In this scenario, molecular analysis has become an important tool for a more precise diagnosis of EB, especially given the recent consolidation and easier access to NGS techniques.^4^, ^5^, ^6^, ^7^, ^8^ Here we compare the results of immunomapping and WES of a Brazilian cohort of 67 patients with EB.

## Method

### Clinical information

We have studied 67 patients from 60 families with different types of EB, all of them attending at Pain and Pediatric Palliative Care Unit, Genetics Unit from Instituto da Criança, and the Department of Dermatology from the Hospital das Clínicas da Faculdade de Medicina da Universidade de São Paulo.

A clinical protocol was elaborated considering familial history, physical and dermatological examination, clinical development, and other major clinical events. Pedigree and photographic registration were collected from all families.

### Immunofluorescence mapping

Immunomapping consists of a technique of indirect immunofluorescence applied upon a fragment of skin containing a fully preserved blister, with analysis of the presence of antigens of the basement membrane zone through the exposition to fluorescent monoclonal antibodies. Just as a reminder, direct immunofluorescence is restricted to cases of acquired autoimmune EB (which were not evaluated in this study) and therefore useless for the patients with hereditary forms of EB.

Most patients already had been submitted to immunomapping at the Immunopathology lab from the present institution prior to this project. Here, the main antibodies used were bullous pemphigoid antibody, laminin antibody, collagen type IV antibody and collagen type VII antibody. Each of these antibodies is found, respectively, in the following sites of the dermoepidermical junction: hemidesmosomes, lamina lucida, lamina densa and sublamina densa.

All immunomapping analysis was performed and reviewed by the head dermatologist of the Immunopathology lab. Finally, the authors point out that 4 families were enlisted in the final stages of the study and therefore not submitted to the immunomapping; also, there were 4 relatives from 3 families in which the index cases already had both immunomapping and molecular results available; therefore, for those 8 patients, only molecular confirmation was performed.

### Molecular analysis and variant classification

Whole-Exome Sequencing (WES) was performed for all 60 families; blood samples were collected from the proband and his/her parents, but preliminary analysis was focused mainly on the affected proband. Following the identification of candidate variant(s) in the index, segregation was then performed through Sanger sequencing in the (overall healthy) parents; whenever there were affected siblings, this approach was performed as well. All the genetic tests were performed by the present study’s partner Japanese researchers in Yokohama.

Variants were interpreted and classified according to the ACMG variant interpretation guidelines (2015). The case was only considered as diagnosed when pathogenic or likely pathogenic variants were observed in a gene that was associated with the phenotype in the studied individual, with compatible zygosity, and in an adequate inheritance pattern.

## Results

### Clinical findings

Typical clinical findings, such as diffuse blistering, were present in most patients (48 of 67). Symptoms and age of appearance were of course variable, but most of them presented typical skin lesions during the first days of life. As expected for an autosomal disease, there was no predominance of gender, with 34 females and 33 males in the studied group. Additionally, there were 4 deaths among these recessive DEB cases, all of them with 20 years or less deceased from sepsis followed by multiple organ failure.

### Dystrophic epidermolysis bullosa (DEB) – *COL7A1*

The vast majority of the patients (47/67) presented variants in the *COL7A1* gene associated with autosomal recessive inheritance; there were only 3 families with recurrence and affected siblings (a total of 7 patients). Given a specific diagnosis of DEB was suggested in only 29 of the 46 patients submitted to immunomapping in this subset (this technique was not available for 5 individuals), concordance with WES was 63% for this type of EB.

According to the literature, the phenotype in these cases tends to be more severe; most patients were born full term with blisters appearing in the first 2 days of life; only two of them were born without fingernails; all of them referred intense itching and other important symptoms were common: chronic pain (78%), malnutrition (71.5%), anemia (48.9%), esophageal stenosis with the need of dilatation (59.6%) and developmental delay (36%). Among them, 3 patients evolved with squamous cell cancer, two of them in the calcaneus region. Unsurprisingly, the 4 deaths among the whole group were from patients with recessive DEB.

On the other hand, 4 of the present patients with DEB presented variants in the *COL7A1* gene associated with an autosomal dominant pattern; all of them were sporadic cases in their respective families. In contrast with the recessive form, clinical findings here can be considered overall mild: blistering did not appear until approximately 2 months of life and was generalized in 3 patients, while confined to hands and feet (acral form) in only one individual; partial nail loss was present, but none patient presented with pseudo syndactyly.

### Epidermolysis bullosa simplex (EBS) – *KRT5/KRT14/PLEC*

Despite being the most common type of EB, it represented the minority of the studied patients, with only 15 patients (12 families); of the 7 different genes associated with this form of the disease, mutations were identified only in the *KRT5*, *KRT14* and *PLEC* genes. Considering the specific diagnosis of EBS was indicated in only 8 of the 12 patients submitted to immunomapping in this group (3 individuals weren’t offered this technique, as they were relatives from the index case), concordance with WES was nearly 67% for this type of EB.

Of the 6 patients with variants in the *KRT5* gene, all of them presented blistering in the first 10 days of life, mainly in extremities and accompanied by chronic pain, itching, and sweating; all presented some degree of nail loss in hands or feet, and one had also fragile teeth, but none of them had esophageal stenosis. There were 4 families with an autosomal dominant form of the disease while 2 others manifested an autosomal recessive pattern; still, there was no significant clinical difference between both patterns of inheritance.

Regarding the 6 patients with variants in *KRT14* gene, all of them also presented blistering in the first days of life and half manifested sweating and anemia during early childhood, but curiously none complained about itching; none had pseudo syndactyly or milia. All of them manifested an autosomal dominant pattern, but 3 were from the same family (father and two sons from different mothers).

Finally, there were 3 patients with variants in the *PLEC* gene, all of whom manifested blisters in the first hours of life; their lesions remained mainly in extremities, with the presence of mild milia, but they evolved with rather normal weight and stature; two of the patients were submitted to esophageal dilatation due to stenosis and one had pseudo syndactyly on her feet. All these families manifested the autosomal recessive form of the disease; two of these families had homozygous mutations, while the remaining one had variants in compound heterozygosis.

### Junctional epidermolysis bullosa (JEB) – *COL17A1*

The only case of JEB was a girl with homozygous variants in *COL17A1* gene, a child of consanguineous parents; since the immunomapping of this isolated case also indicated JEB, correspondence of both techniques for this form of the disease was 100% for this type of EB. The patient’s phenotype could be characterized as moderate; she manifested blisters since her third day of life and also referred to chronic pains and constant itching. At 16 years old, the patient had difficulty walking, accompanied by mild anemia and malnutrition; her dermatological exam revealed blisters located mainly in crease areas (arms, knees, feet), without milia, and her hair was thin and fragile; notably, her toes’ nails were absent.

### Genetic findings

The authors have studied 67 patients from 60 families with different types of EB, all of them attending the institution’s hospital.

In this scenario, there were: 47 patients (43 families) with recessive DEB, 4 patients with dominant DEB, 15 patients (12 families) with EBS and 1 patient with JEB. From the 60 different variants in genes related to EB observed in the studied patients, 15 have never been published in the literature to our knowledge, with 10 being associated with recessive DEB, 3 with dominant DEB and 2 with EBS; most of them were null mutations (*nonsense*, *frameshift* or canonical splice site variants), with only 3 being *missense* variants. Details are described in the supplementary materials.

### Immunomapping

Immunomapping was available in 59 of these patients; 8 of the total 67 patients could not proceed to skin biopsy investigation. There was concordance between immunomapping and exome results in 37 (62%) of these cases, while in 13 (22%) immunomapping was proven wrong by molecular analysis; additionally, there were 9 patients (15%) in which immunomapping results were inconclusive, as shown in Table 1, Table 2. Therefore, a total of 22 patients’ immunomapping (37%) established either wrong or no diagnosis at all.

**Table 1:** General comparison of diagnostic confirmation between WES and immunomapping in patients with EB.

Whole-Exome sequencing (WES)	Immunomapping (IM)
59/59 patients confirmed (100%)	37/59 patients confirmed (62.7%)

**Table 2:** Detailed comparison between results of WES and immunomapping in patients with EB.

Family	Patient	Genetic etiology (WES)	Immunomapping	Match
1	1	Dystrophic EB AR (*COL7A1*)	Dystrophic EB AR	+
	2	Dystrophic EB AR (*COL7A1*)	Inconclusive	NA
	3	Dystrophic EB AR (*COL7A1*)	Inconclusive	NA
2	4	Dystrophic EB AR (*COL7A1*)	Dystrophic EB AD	–
	5	Dystrophic EB AR (*COL7A1*)	Not performed	NA
3	6	Dystrophic EB AR (*COL7A1*)	Dystrophic EB AD	+
	7	Dystrophic EB AR (*COL7A1*)	Dystrophic EB AD	–
4	8	Dystrophic EB AR (*COL7A1*)	Dystrophic EB AR	+
5	9	Dystrophic EB AR (*COL7A1*)	Dystrophic EB AR	+
6	10	Dystrophic EB AR (*COL7A1*)	Dystrophic EB AR	+
7	11	Dystrophic EB AR (*COL7A1*)	Dystrophic EB AR	+
8	12	Dystrophic EB AR (*COL7A1*)	Dystrophic EB AR	+
9	13	Dystrophic EB AR (*COL7A1*)	Dystrophic EB AR	+
10	14	Dystrophic EB AR (*COL7A1*)	Dystrophic EB AR	+
11	15	Dystrophic EB AR (*COL7A1*)	Dystrophic EB AR	+
12	16	Dystrophic EB AR (*COL7A1*)	Dystrophic EB AR	+
13	17	Dystrophic EB AR (*COL7A1*)	Dystrophic EB AR	+
14	18	Dystrophic EB AR (*COL7A1*)	Dystrophic EB AR	+
15	19	Dystrophic EB AR (*COL7A1*)	Dystrophic EB AR	+
16	20	Dystrophic EB AR (*COL7A1*)	Dystrophic EB AR	+
17	21	Dystrophic EB AR (*COL7A1*)	Dystrophic EB AR	+
18	22	Dystrophic EB AR (*COL7A1*)	Dystrophic EB AR	+
19	23	Dystrophic EB AR (*COL7A1*)	Dystrophic EB AR	+
20	24	Dystrophic EB AR (*COL7A1*)	Dystrophic EB AR	+
21	25	Dystrophic EB AR (*COL7A1*)	Dystrophic EB AR	+
22	26	Dystrophic EB AR (*COL7A1*)	Dystrophic EB AR	+
23	27	Dystrophic EB AR (*COL7A1*)	Dystrophic EB AR	+
24	28	Dystrophic EB AR (*COL7A1*)	Dystrophic EB AR	+
25	29	Dystrophic EB AR (*COL7A1*)	Dystrophic EB AR	+
26	30	Dystrophic EB AR (*COL7A1*)	Dystrophic EB AD	–
27	31	Dystrophic EB AR (*COL7A1*)	Dystrophic EB AD	–
28	32	Dystrophic EB AR (*COL7A1*)	Dystrophic EB AD	–
29	33	Dystrophic EB AR (*COL7A1*)	Dystrophic EB AD	–
30	34	Dystrophic EB AR (*COL7A1*)	Dystrophic EB AD	–
31	35	Dystrophic EB AR (*COL7A1*)	Dystrophic EB AD	–
32	36	Dystrophic EB AR (*COL7A1*)	Dystrophic EB AD	–
33	37	Dystrophic EB AR (*COL7A1*)	Junctional EB	–
34	38	Dystrophic EB AR (*COL7A1*)	Dystrophic EB AD	–
35	39	Dystrophic EB AR (*COL7A1*)	Inconclusive	NA
36	40	Dystrophic EB AR (*COL7A1*)	Inconclusive	NA
37	41	Dystrophic EB AR (*COL7A1*)	Inconclusive	NA
38	42	Dystrophic EB AR (*COL7A1*)	Inconclusive	NA
39	43	Dystrophic EB AR (*COL7A1*)	Not performed	NA
40	44	Dystrophic EB AR (*COL7A1*)	Dystrophic EB (without specification)	+
41	45	Dystrophic EB AR (*COL7A1*)	Not performed	NA
42	46	Dystrophic EB AR (*COL7A1*)	Not performed	NA
43	47	Dystrophic EB AR (*COL7A1*)	Not performed	NA
44	48	Dystrophic EB AD (*COL7A1*)	Dystrophic EB AD	+
45	49	Dystrophic EB AD (*COL7A1*)	Dystrophic EB AD	+
46	50	Dystrophic EB AD (*COL7A1*)	Dystrophic EB AD	+
47	51	Dystrophic EB AD (*COL7A1*)	Dystrophic EB AD	+
48	52	EB simplex AD (*KRT5*)	EB simplex	+
	53	EB simplex AD (*KRT5*)	Not performed	NA
49	54	EB simplex AR (*KRT5*)	EB simplex	+
50	55	EB simplex AR (*KRT5*)	EB simplex	+
51	56	EB simplex AD (*KRT5*)	EB simplex	+
52	57	EB simplex AD (*KRT5*)	Inconclusive	NA
53	58	EB simplex AD (*KRT14*)	EB simplex	+
	59	EB simplex AD (*KRT14*)	Not performed	NA
	60	EB simplex AD (*KRT14*)	Not performed	NA
54	61	EB simplex AD (*KRT14*)	EB simplex	+
55	62	EB simplex AD (*KRT14*)	EB simplex	+
56	63	EB simplex AD (*KRT14*)	Dystrophic EB AD	–
57	64	EB simplex AR (*PLEC*)	EB simplex	+
58	65	EB simplex AD (*PLEC*)	Inconclusive	NA
59	66	EB simplex AR (*PLEC*)	Inconclusive	NA
60	67	Junctional EB (*COL17A1*)	Junctional EB	+

AD, Autosomal Dominant; AR, Autosomal Recessive; NA, Not Available.

Finally, the authors performed a quantified agreement analysis regarding the immunomapping of the 50 patients who received either positive (38) or negative (12) results, when compared with the WES results; even though the Kappa test tends to underestimate the level of agreement towards rare diseases, it was equal to -0,261 (95% Confidence Interval, from -0,388 to -0,135) in this case, indicating no agreement between these two exams.

## Discussion

There was a clear predominance of DEB (associated with a mutation in *COL7A1* gene), with 51 of the 67 patients (76%) presenting this type of disease, which usually represents a more severe phenotype, especially in the autosomal recessive form. Considering that the patients in this study were attending the group of Pain and Palliative Care, an elevated frequency of severeness was already expected; still, the total count of deaths under 20 years stands apart from the reported Brazilian and global death rates, perhaps indicating the lack of proper intense care support for these patients in some local hospitals. Other than that, phenotypes were similar to the ones described in the literature for each respective gene.

Regarding familiar history, it is important to point out that it was somewhat biased, given that 11 of the 60 families presented some level of consanguinity; notoriously, 8 of these cases presented homozygous mutations in the *COL7A1* gene. Still, autosomal recessive forms of the disease were the majority even in the families without consanguinity, with 41 families presenting variants in compound heterozygosis (meaning the patient received a different deleterious variant in the same gene from each health parent). As expected for autosomal dominant forms, there was a majority of 6 cases of *de novo* mutations in heterozygosis, not inherited from either parent; vertical transmission was observed in only 2 families, meaning that the child and its father or mother were both affected.

In earlier times, electronic microscopic analysis had been the former gold standard for EB diagnosis, but its expressive costs and difficulty of access (few centers have this equipment at disposition and perhaps even fewer people have experience using it) gradually determined its decline.

As mentioned before, until recently the gold standard for diagnosis has been immunomapping through skin biopsy. Even though it can usually identify the cutaneous level in which the cleavage of the blisters resides (intradermal or subepidermal), it is, however, unable to indicate with certainty the affected gene and, thus, the inheritance pattern of each patient. Besides that, it is an invasive procedure (with the need for local anesthesia and subsequent sutures) and its results are rarely inconclusive, with often a need for a second skin biopsy, which can be particularly problematic in EB patients.

Nowadays, molecular analysis has become an important tool for a more precise diagnosis of EB. Unsurprisingly, its use has been strongly recommended not only due to its precision and potential lesser cost but also because it would avoid the harm of damaging the skin in such vulnerable patients since it works fine with just one drop of blood or even a spit of saliva from the patient.

Nevertheless, it is important to point out a few disadvantages regarding the genetic diagnosis of EB. First of all, more generalized methods and insufficient resources dedicated to molecular testing, particularly in distant regions. Other than that, there is the fact that there are at least 18 genes associated with the various types of EB, with over 1000 different mutations described; previous approaches with Sanger sequencing of one gene or even specific known mutations could not warrant confirmation of etiology, often representing a long and more expensive path to diagnosis.

Fortunately, the advances in NGS technique can now provide simultaneous analysis of one or hundreds of genes not only with the same cost but also in a much faster and more automatic way; besides, the possibility of collecting blood samples in primary health centers or even the use saliva kits at home, both which can be rather simply sent through the mail to reference facilities, represents an obvious expansion of the range of testing.

Here it is important to point out that Whole-Exome Sequencing (WES) analyzes not only genes related with EB, but actually all human genes. This of course represents a higher cost for the test when compared to a panel, but still, in the authors’ experience, it can often be more difficult for laboratories to establish a cheaper focused NGS panel for one single condition rather than offering WES for several patients with various genetic hypothesis (from intellectual disability to short stature). Considering all that, even though the present study used WES for genetic analysis, the authors believe these results can be applied to all NGS techniques since germline mutations in genes related to EB would be detected by either WES or a focused NGS panel.

Despite being considered more expensive than immunomapping at first, it is important to express that NGS costs have kept constantly fallen over the last years. The potential cost-effectiveness of the NGS panel focused in genes related with EB for diagnosis in a country with continental dimensions such as Brazil has already been suggested by another recent study.^7^

At last, the potential application of gene therapy in a rare and, so far, incurable disable such as EB cannot be undermined. Despite the numerous challenges on this path, more than one research has shown promising results in the use of these new therapeutic approaches^9^, ^10^, ^11^ in patients with different types of EB. As the knowledge of the genotype is absolutely necessary for such selected cases, once again the authors highlight that immunomapping alone would provide insufficient information in order to decide if each patient is eligible or not for specific treatment. On a final note, considering the growing availability and demand of IVF (*in vitro* fertilization),^12^ genetic diagnosis of EB also becomes all the more necessary.

## Conclusion

EB contemplates a group of genetic skin diseases that are marked by severe fragility and recurrent blistering. As a rare congenital disorder caused by mutations in multiple genes, proper diagnosis can be challenging for a number of reasons.

Although immunomapping has been useful in services where molecular studies are not available, genetic analysis performed through NGS has shown a higher tax of diagnosis. Besides, all positive results will lead to the confirmation of the inheritance pattern of each family, which often immunomapping cannot predict. Additionally, in the not-so-distant future, knowledge of the genotype will be necessary in order to decide if each patient is eligible or not for specific forms of gene therapy, and also assisted reproduction techniques.

Finally, we conclude that the molecular study (either with NGS focused panel or WES) is cost-effective for the diagnosis of EB, not only due to its high efficiency and accuracy but also because it eliminates the need for skin biopsy. Thus, we consider it will provide the patients overall better prognosis, prenatal genetic diagnosis, and genetic counseling.

## Financial support

Kim CA CNPQ 304897/2020-5; FAPESP 2019/21644-0.

Matsumoto N - Supported by the Japan Agency for Medical Research and Development (AMED) [grant numbers JP22ek0109486, JP22ek0109549, JP22ek0109493]; the Takeda Science Foundation.

## Authors' contributions

Samantha Vernaschi Kelmann: Contributed largely to the conception and design of the article, as well as the dermatological evaluation and interpretation of data.

Bruno de Oliveira Stephan: Contributed largely to the conception and design of the article, as well as the genetic analysis and interpretation of data.

Silvia Maria de Macedo Barbosa: Contributed to the clinical follow-up and management of patients.

Rita Tiziana Verardo Polastrini: Contributed to the clinical follow-up and management of patients.

Zilda Najjar Prado de Oliveira: Contributed to the execution and interpretation of immunomapping.

Maria Cecília Rivitti-Machado: Contributed to the execution and interpretation of immunomapping.

Gustavo Marquezani Spolador: Contributed to the genetic counseling and management of patients.

Rachel Sayuri Honjo: Contributed to the draft and critical revision of the article.

Ken Saida: Contributed to the execution and interpretation of the molecular testing.

Naomichi Matsumoto: Contributed to the execution and interpretation of the molecular testing.

Chong Ae Kim: Contributed to the draft and critical revision of the article.

## Conflicts of interest

None declared.
