# Supplementary material for: Advantages of whole-exome sequencing over immunomapping in 67 Brazilian patients with epidermolysis bullosa
Source: An Bras Dermatol. 2024 Feb 16;99(3):350–6. doi: 10.1016/j.abd.2023.07.002 (PMC11074559; doi:10.1016/j.abd.2023.07.002)
Supplement: Supplementary file 1 [file mmc1.docx]

**ABD-D-23-00228 - Supplementary Materials**

**Supplementary Table 1** Results from patients with variants in the *COL7A1* gene (autosomal recessive).

| **Family** | **Patient** | **Sex** | **Variants identified** | **Literature** | **Status** | **Consanguinity** |
| --- | --- | --- | --- | --- | --- | --- |
| 1 | 1 | M | NM_000094: c.5047C>T: p.(Arg1683*) | Known mutation (rs760063197) | Homozygosity | No |
|  | 2 | M | NM_000094: c.5047C>T: p.(Arg1683*) | Known mutation (rs760063197) | Homozygosity | No |
|  | 3 | M | NM_000094: c.5047C>T: p.(Arg1683*) | Known mutation (rs760063197) | Homozygosity | No |
| 2 | 4 | F | NM_000094: c.2171del: p.(Gly724Alafs*30) | Known mutation (rs1336602322) | Compound heterozygosity | No |
|  |  |  | NM_000094: c.887del: p.(Gly296Valfs*5) | Known mutation (rs1131691385) |  | No |
|  | 5 | M | NM_000094: c.2171del: p.(Gly724Alafs*30) | Known mutation (rs1336602322) | Compound heterozygosity | No |
|  |  |  | NM_000094: c.887del: p.(Gly296Valfs*5) | Known mutation (rs1131691385) |  | No |
| 3 | 6 | F | NM_000094: c.6502-2A>G | Known mutation (rs773265287) | Compound heterozygosity | No |
|  |  |  | NM_000094: c.6182G>A:p.(Gly2061Glu) | Known mutation |  | No |
|  | 7 | F | NM_000094: c.6502-2A>G | Known mutation (rs773265287) | Compound heterozygosity | No |
|  |  |  | NM_000094: c.6182G>A: p.(Gly2061Glu) | Known mutation |  | No |
| 4 | 8 | F | NM_000094: c.58_70del: p.(Arg20Serfs*6) | Known mutation (rs2045987838) | Homozygosity | No |
| 5 | 9 | F | NM_000094: c.6528dup: p.(Gly2177Trpfs*113) | Known mutation (rs768128088) | Homozygosity | Yes |
| 6 | 10 | F | NM_000094: c.5047C>T: p.(Arg1683*) | Known mutation (rs760063197) | Homozygosity | No |
| 7 | 11 | F | NM_000094: c.7249C>T: p.(Gln2417*) | Known mutation (rs983476178) | Compound heterozygosity | Yes |
|  |  |  | NM_000094: c.4018C>T: p.(Arg1340*) | Known mutation (rs761927109) |  |  |
| 8 | 12 | F | NM_000094: c.5572G>T: p.Gln1858* | Novel mutation | Homozygosity | Yes |
| 9 | 13 | M | NM_000094: c.4018C>T: p.(Arg1340*) | Known mutation (rs761927109) | Homozygosity | No |
| 10 | 14 | M | NM_000094: c.7757C>T: p.(Gln2586*) | Novel mutation | Homozygosity | Yes |
| 11 | 15 | M | NM_000094: c.6081dup: p.(Gly2028Argfs*71) | Known mutation (rs780623622) | Homozygosity | Yes |
| 12 | 16 | M | NM_000094: c.6527dup: p.(Gly2177Trpfs*113) | Known mutation (rs768128088) | Compound heterozygosity | No |
|  |  |  | NM_000094: c.5047C>T: p.(Arg1683*) | Known mutation (rs760063197) |  |  |
| 13 | 17 | M | NM_000094: c.7344G>A: p.(Val2448=) | Known mutation (rs201728948) | Compound heterozygosity | No |
|  |  |  | NM_000094: c.5047C>T: p.(Arg1683*) | Known mutation (rs760063197) |  |  |
| 14 | 18 | M | NM_000094: c.6022C>T: p.(Arg2008Cys) | Known mutation (rs1055680335) | Compound heterozygosity | No |
|  |  |  | NM_000094: c.425A>G: p.(Lys142Arg) | Known mutation (rs121912856) |  |  |
| 15 | 19 | F | NM_000094: c.5132_5133insTCACC: p.(Gly1712Hisfs*131) | Known mutation (rs770456964) | Compound heterozygosity | No |
|  |  |  | NM_000094: c.58_70del: p.(Arg20Serfs*6) | Known mutation (rs2045987838) |  |  |
| 16 | 20 | M | NM_000094: c.7078G>A: p.(Gly2360Arg) | Known mutation (rs916512411) | Compound heterozygosity | No |
|  |  |  | NM_000094: c.6527dup: p.(Gly2177Trpfs*113) | Known mutation (rs768128088) |  |  |
| 17 | 21 | M | NM_000094: c.6527dup: p.(Gly2177Trpfs*113) | Known mutation (rs768128088) | Compound heterozygosity | No |
|  |  |  | NM_000094:c.6040C>T: p.(Gln2014*) | Novel mutation |  |  |
| 18 | 22 | F | NM_000094: c.6134del: p.(Pro2045Glnfs*161) | Novel mutation | Compound heterozygosity | No |
|  |  |  | NM_000094: c.5132_5133insTCACC: p.(Gly1712Hisfs*131) | Known mutation (rs770456964) |  |  |
| 19 | 23 | M | NM_000094: c.2783_2784insGACAC: p.(Gln929Thrfs*6) | Novel mutation | Compound heterozygosity | No |
|  |  |  | NM_000094: c.325_326insCG: p.(Glu109Alafs*39) | Known mutation (rs1235811820) |  |  |
| 20 | 24 | F | NM_000094: c.4378C>T: p.(Gln1460*) | Novel mutation | Compound heterozygosity | No |
|  |  |  | NM_000094: c.1758del: p.(Ser587Valfs*28) | Known mutation (rs2045631974) |  |  |
| 21 | 25 | M | NM_000094: c.7828C>T: p.(Arg2610*) | Known mutation (rs1064793916) | Compound heterozygosity | No |
|  |  |  | NM_000094: c.4018C>T: p.(Arg1340*) | Known mutation (rs761927109) |  |  |
| 22 | 26 | F | NM_000094: c.2784_2785insGACAC: p.(Gln929Aspfs*6) | Novel mutation | Compound heterozygosity | No |
|  |  |  | NM_000094: c.5134_5135insTCACC: p.(Gly1712Serfs*131) | Known mutation (rs770456964) |  |  |
| 23 | 27 | F | NM_000094: c.7474C>T: p.(Arg2492*) | Known mutation (rs765529435) | Compound heterozygosity | No |
|  |  |  | NM_000094: c.5047C>T: p.(Arg1683*) | Known mutation (rs760063197) |  |  |
| 24 | 28 | F | NM_000094: c.5047C>T: p.(Arg1683*) | Known mutation (rs760063197) | Compound heterozygosity | No |
|  |  |  | NM_000094: c.676C>T: p.(Arg226*) | Known mutation (rs753819164) |  |  |
| 25 | 29 | M | NM_000094: c.6528dup: p.(Gly2177Trpfs*113) | Known mutation (rs768128088) | Homozygosity | No |
| 26 | 30 | M | NM_000094: c.6716G>A: p.(Gly2239Asp) | Novel mutation | Compound heterozygosity | No |
|  |  |  | NM_000094:c.2783_2784insGACAC: p.(Gln929Thrfs*6) | Novel mutation |  |  |
| 27 | 31 | F | NM_000094: c.4678G>A: p.(Gly1560Arg) | Known mutation (rs2044774392) | Compound heterozygosity | No |
|  |  |  | NM_000094: c.657del: p.(Gly220Valfs*5) | Known mutation (rs1575494051)) |  |  |
| 28 | 32 | F | NM_000094: c.8304+1G>A | Known mutation (rs759579761) | Compound heterozygosity | No |
|  |  |  | NM_000094: c.5047C>T: p.(Arg1683*) | Known mutation (rs760063197) |  |  |
| 29 | 33 | M | NM_000094: c.5018G>A: p.(Gly1673Glu) | Known mutation | Compound heterozygosity | No |
|  |  |  | NM_000094: c.4183_4189dup: p.(Ala1397Glyfs*7) | Novel mutation |  |  |
| 30 | 34 | F | NM_000094: c.6022C>T: p.(Arg2008Cys) | Known mutation (rs1055680335) | Compound heterozygosity | No |
|  |  |  | NM_000094: c.5047C>T: p.(Arg1683*) | Known mutation (rs760063197) |  |  |
| 31 | 35 | M | NM_000094: c.6082G>A: p.(Gly2028Arg) | Known mutation (rs762162799) | Compound heterozygosity | No |
|  |  |  | NM_000094: c.4018C>T: p.(Arg1340*) | Known mutation (rs761927109) |  |  |
| 32 | 36 | M | NM_000094: c.7249C>T: p.(Gln2417*) | Known mutation (rs983476178) | Compound heterozygosity | No |
|  |  |  | NM_000094: c.8245G>A: p.(Gly2749Arg) | Known mutation (rs121912853) |  |  |
| 33 | 37 | F | NM_000094: c.7380+2T>C | Known mutation (rs1249145909) | Compound heterozygosity | No |
|  |  |  | NM_000094: c.6527dup: p.(Gly2177Trpfs*113) | Known mutation (rs768128088) |  |  |
| 34 | 38 | M | NM_000094: c.7078G>A: p.(Gly2360Arg) | Known mutation (rs916512411) | Homozygosity | Yes |
| 35 | 39 | M | NM_000094: c.58_70del: p.(Arg20Serfs*6) | Known mutation (rs2045987838) | Homozygosity | No |
| 36 | 40 | F | NM_000094: c.5047C>T: p.(Arg1683*) | Known mutation (rs760063197) | Compound heterozygosity | No |
|  |  |  | NM_000094: c.4018C>T: p.(Arg1340*) | Known mutation (rs761927109) |  |  |
| 37 | 41 | M | NM_000094: c.706C>T:p.(Arg236*) | Known mutation (rs121912854) | Compound heterozygosity | No |
|  |  |  | NM_000094: c.325_326insCG:p.(Glu109Alafs*39) | Known mutation (rs1235811820) |  |  |
| 38 | 42 | M | NM_000094: c.7222C>T: p.(Gln2408*) | Novel mutation | Compound heterozygosity | No |
|  |  |  | NM_000094: c.5047C>T: p.(Arg1683*) | Known mutation (rs760063197) |  |  |
| 39 | 43 | F | NM_000094: c.4463del: p.(Leu1488Argfs*222) | Novel mutation | Homozygosity | Yes |
| 40 | 44 | M | NM_000094: c.4613G>A: p.(Arg1538His) | Known mutation (rs2229824)* | Compound heterozygosity | No |
|  |  |  | NM_000094: c.4018C>T: p.(Arg1340*) | Known mutation (rs761927109) |  |  |
| 41 | 45 | M | NM_000094: c.1080G>A: p.(Trp360*) | Known mutation (rs375604839) | Compound heterozygosity | No |
|  |  |  | NM_000094: c.887del: p.(Gly296Valfs*5) | Known mutation (rs1131691385) |  |  |
| 42 | 46 | M | NM_000094: c.5047C>T: p.(Arg1683*) | Known mutation (rs760063197) | Homozygosity | Yes |
| 43 | 47 | F | NM_000094: c.8109+1G>A | Known mutation (rs1050797523) | Compound heterozygosity | No |
|  |  |  | NM_000094: c.4018C>T: p.(Arg1340*) | Known mutation (rs761927109) |  |  |

**Supplementary Table 2** Results from patients with variants in the *COL7A1* gene (autosomal dominant).

| **Family** | **Patient** | **Sex** | **Variants identified** | **Literature** | **Status** |
| --- | --- | --- | --- | --- | --- |
| 44 | 48 | M | NM_000094: Gly2034_Gly2040del | Novel mutation | Heterozygosity *(De novo)* |
| 45 | 49 | F | NM_000094: c.6110G>A: p.(Gly2037Glu) | Known mutation (rs121912846) | Heterozygosity *(De novo)* |
| 46 | 50 | F | NM_000094: c.6218G>A: p.(Gly2073Asp) | Novel mutation | Heterozygosity *(De novo)* |
| 47 | 51 | F | NM_000094: c.6026G>T: p.(Gly2009Val) | Novel mutation | Heterozygosity *(De novo)* |

**Supplementary Table 3** Results from patients with variants in the *KRT5/KRT14/PLEC* genes.

| **Family** | **Patient** | **Sex** | **Gene** | **Variants identified** | **Literature** | **Status** | **Inheritance** |
| --- | --- | --- | --- | --- | --- | --- | --- |
| 48 | 52 | F | *KRT5* | NM_000424: c.555+1G>A | Known mutation (rs886039403) | Heterozygosity | AD |
| 48 | 53 | F | *KRT5* | NM_000424: c.555+1G>A | Known mutation (rs886039403) | Heterozygosity | AD |
| 49 | 54 | F | *KRT5* | NM_000424: c.598T>C:p.(Trp200Arg) | Known mutation | Compound heterozygosity | AR |
|  |  |  |  | NM_000424: c.770+2T>A | Novel mutation |  |  |
| 50 | 55 | M | *KRT5* | NM_000424: c.598T>C:p.(Trp200Arg) | Known mutation | Homozygosity | AR |
| 51 | 56 | F | *KRT5* | NM_000424: c.449T>C:p.(Leu150Pro) | Known mutation (rs62635291) | Heterozygosity *(De novo)* | AD |
| 52 | 57 | F | *KRT5* | c.1429G>A:p.(Glu477Lys) | Known mutation (rs59190510) | Heterozygosity *(De novo)* | AD |
| 53 | 58 | M | *KRT14* | NM_000526: c.373C>G:p.(Arg125Gly) | Known mutation (rs60399023) | Heterozygosity | AD |
| 53 | 59 | M | *KRT14* | NM_000526: c.373C>G:p.(Arg125Gly) | Known mutation (rs60399023) | Heterozygosity | AD |
| 53 | 60 | M | *KRT14* | NM_000526: c.373C>G:p.(Arg125Gly) | Known mutation (rs60399023) | Heterozygosity | AD |
| 54 | 61 | F | *KRT14* | NM_000526: c.374G>A:p.(Arg125His) | Known mutation (rs58330629) | Heterozygosity *(De novo)* | AD |
| 55 | 62 | F | *KRT14* | NM_000526: c.373C>T:p.(Arg125Cys) | Known mutation (rs60399023) | Heterozygosity *(De novo)* | AD |
| 56 | 63 | M | *KRT14* | NM_000526: c.1243T>C:p.(Tyr415His) | Known mutation (rs58380626) | Heterozygosity | AD |
| 57 | 64 | F | *PLEC* | NM_000445: c.7393C>T:(p.Arg2465*) | Known mutation (rs1554690016) | Homozygosity | AR |
| 58 | 65 | F | *PLEC* | NM_000445: c.5470C>T:p.(Arg1824Cys) | Known mutation (rs782175669) | Compound heterozygosity | AR |
|  |  |  |  | NM_000445: c.9976C>T: p.(Arg3326Trp) | Known mutation (rs142805337)* |  |  |
| 59 | 66 | M | *PLEC* | NM_000445: c.4543C>T:p.(Gln1515*) | Novel mutation | Homozygosity | AR |

**Supplementary Table 4** Results from patient with variant in the *COL17A1* gene:

| **Family** | **Patient** | **Sex** | **Variants identified** | **Literature** | **Status** |
| --- | --- | --- | --- | --- | --- |
| 60 | 67 | F | NM_000494: c.2383C>T:p.(Arg795*) | Known mutation (rs1277370326) | Homozygosity |
